# Flavivirus NS3 and NS5 proteins interaction network: a high-throughput yeast two-hybrid screen

**DOI:** 10.1186/1471-2180-11-234

**Published:** 2011-10-20

**Authors:** Marc Le Breton, Laurène Meyniel-Schicklin, Alexandre Deloire, Bruno Coutard, Bruno Canard, Xavier de Lamballerie, Patrice Andre, Chantal Rabourdin-Combe, Vincent Lotteau, Nathalie Davoust

**Affiliations:** 1 Inserm Unit 851, Lyon, France; 2 Université de Lyon, SFR BioSciences Gerland-Lyon Sud, Lyon, France; 3 Architecture et Fonction des Macromolécules Biologiques, CNRS and Universités d'Aix-Marseille I et II, UMR 6098, Marseille, France; 4 UMR190, IRD and Université d'Aix Marseille II, Marseille, France; 5 Hospices Civils de Lyon, Hôpital de la Croix-Rousse, Laboratoire de virologie, Lyon, France; 6 Ecole Normale Supérieure de Lyon, Lyon, France

## Abstract

**Background:**

The genus *Flavivirus *encompasses more than 50 distinct species of arthropod-borne viruses, including several major human pathogens, such as West Nile virus, yellow fever virus, Japanese encephalitis virus and the four serotypes of dengue viruses (DENV type 1-4). Each year, flaviviruses cause more than 100 million infections worldwide, some of which lead to life-threatening conditions such as encephalitis or haemorrhagic fever. Among the viral proteins, NS3 and NS5 proteins constitute the major enzymatic components of the viral replication complex and are essential to the flavivirus life cycle.

**Results:**

We report here the results of a high-throughput yeast two-hybrid screen to identify the interactions between human host proteins and the flavivirus NS3 and NS5 proteins. Using our screen results and literature curation, we performed a global analysis of the NS3 and NS5 cellular targets based on functional annotation with the Gene Ontology features. We finally created the first flavivirus NS3 and NS5 proteins interaction network and analysed the topological features of this network. Our proteome mapping screen identified 108 human proteins interacting with NS3 or NS5 proteins or both. The global analysis of the cellular targets revealed the enrichment of host proteins involved in RNA binding, transcription regulation, vesicular transport or innate immune response regulation.

**Conclusions:**

We proposed that the selective disruption of these newly identified host/virus interactions could represent a novel and attractive therapeutic strategy in treating flavivirus infections. Our virus-host interaction map provides a basis to unravel fundamental processes about flavivirus subversion of the host replication machinery and/or immune defence strategy.

## Background

The family of *Flaviviridae *contains three genera, *Pestivirus, Hepacivirus *and *Flavivirus*. The genus *Flavivirus *is subdivided into more than 50 distinct species of arthropod-borne viruses including major human pathogens, such as West Nile (WNV), yellow fever (YFV), Japanese encephalitis (JEV) and the four serotypes of dengue viruses (DENV types 1-4) [1]. A number of flavivirus infections may lead to acute lethal haemorrhagic fever or encephalitis in patients and are therefore of great global public health concern. Flaviviruses are enveloped viruses with a single-stranded, non-segmented positive RNA genome [2]. The approximate 11 kb long genome contains only one open reading frame encoding a single polyprotein, which is thereafter cleaved by cellular and viral proteases to form three structural and seven non-structural proteins (NS1, NS2a, NS2b, NS3, NS4a, NS4b, NS5). Recent studies also reported that a NS1' viral protein, which is often detected during infection, is the possible result of ribosomal frameshifting [3]. The NS3 protein has a pivotal function in flavivirus RNA replication and viral protein maturation [4,5]. It consists of two functional domains, protease and helicase in N-and C-terminus, respectively. NS5 protein is constituted by two distinct domains as well, namely an N-terminal methyltransferase and a C-terminal RNA-dependent RNA polymerase that are required for capping and synthesis of the viral RNA genome, respectively [6]. NS3 and NS5 proteins are the major enzymatic components of the viral replication complex, which promotes efficient viral replication in close association with cellular host factors [7]. Due to their numerous functions and their central role in the virus life cycle, NS3 and NS5 have been designated as important drug targets [8,9].

To identify host factors interacting with flavivirus NS3 and NS5 proteins, we have conducted a high-throughput yeast two-hybrid (Y2H) screen. Since the pioneer study published by Uetz *et al*. in 2006 on Herpes viruses interactome, the use of the high-throughput yeast two-hybrid (Y2H) technique to conduct genome-scale screens of virus-host protein interactions has led to major advances in our understanding of viral infections [10-13]. These results from the integrative system biology approaches highlighted the ability of viral proteins to interfere with intracellular pathways to the benefit of viral replication. Indeed, viruses not only take advantage of such interactions for their replication or to escape host defense but also induce cellular interactome perturbations leading eventually to infection-related diseases. Recently, studies using genome-wide RNA interference screens in human or insect cells were able to provide the identification of numerous host cell factors potentially required to interfere with DENV or WNV infection [14]. Some of the targets identified are host (mammalian) or vector (insect) exclusive, others are common to both. This suggests that conservation of required factors between dipteran and human hosts is associated to flavivirus propagation [15]. These studies also identified host factors specific to either WNV or DENV, suggesting that the mechanisms used to interact with host cells can be either virus specific or conserved between several members of the genus *Flavivirus *[16].

We report here the identification of 108 human proteins that interact with flavivirus NS3 or NS5 proteins or both. Based on our Y2H screen results, we created the first flavivirus NS3 and NS5 proteins interaction network composed of 186 interactions and involving 120 distinct human proteins. Analysis of this virus-host interaction network revealed the topological features of the cellular proteins targeted by the flavivirus NS3 and NS5 proteins and identified functional pathways related to flavivirus biology.

## Methods

### Plasmid DNA contructs

Coding sequences for NS3 and NS5 *Flaviviruses *full-length proteins or NS3 helicase, NS3 protease, NS5 polymerase and NS5 methyltransferase functional domains were provided in pDONR207 entry vector (Gateway, Invitrogen) by Bruno Coutard (Architecture et Fonction des Macromolécules Biologiques, UMR6098, Marseille) and referenced in ViralORFeome database [17]. The viral ORFs were isolated from the following viruses: dengue virus serotype 1 (strain D1/H/IMTSSA/98/606), Alkhurma virus (strain 1176), West Nile virus (Strain paAn001), Japanese Encephalitis virus (strain Beijing1), Kunjin virus (MRM61C) and Tick borne encephalitis virus (strain 263). Cellular ORF coding for AZI2 was purchased from Invitrogen (clone IOH41551) and coding sequences for NFKBIA, and TRAF4 were obtained from the Human ORF Collection (OHS4187, Open Biosystems). Viral and cellular coding sequences were subsequently transferred by *in vitro *recombination from pDONR207 into different Gateway-compatible destination vectors following manufacturer's recommendation (LR cloning reaction, Invitrogen). To perform yeast-two hybrid experiments, human prey coding sequences were recombined into pACT2 (Invitrogen) to be expressed in fusion downstream of the activation domain of Gal4 (Gal4-AD) and viral bait coding sequences into pGBKT7 to be expressed in fusion downstream of the DNA binding domain of Gal4 (Gal4-BD). In mammalian cells, GST-tag and 3xFLAG-tag fusions were achieved using pDEST27 (Invitrogen), or pCI-neo-3XFLAG (kindly provided by Y. Jacob Institut Pasteur) vectors, respectively.

### Yeast two-hybrid assay

Viral cDNAs cloned into bait Gal4-BD vector pGBKT7, were transformed into AH109 yeast strain (Clontech) and used to screen by mating human cDNA libraries from liver, brain, spleen and bronchial epithelia cloned in the GAL4-AD pACT2 vectors, and transformed into prey Y187 yeast strains. The mating between baits and prey yeast cells was performed on a selective medium lacking histidine and supplemented with 10 mM 3-amino-triazole (3-AT; Sigma-Aldrich). After 6 days of culture on selective medium, [His+] diploids colonies were isolated and further selected over 3 weeks by culture on selective medium to eliminate false-positives colonies. After selection, yeast colonies were treated with zymolyase in order to digest their cell walls, and AD-cDNAs were amplified by PCR using primers that hybridize within the pACT2 regions flanking cDNA inserts (Fwd: gacggaccaaactgcgtataacg, Rev: ccaaacctctggcgaagaagtcc). PCR products were sequenced (GATC Biotech) and cellular interactors were identified by BLAST analysis as previously described [18].

### Literature curation of interactions between flavivirus and cellular proteins

Interactions retrieved from literature, describing binary interactions between cellular and flavivirus proteins, were extracted from VirHostNet knowledge base [19] after PubMed extensive curation. Briefly, VirHostNet is an up to date knowledge base for the management and the analysis of proteome-wide virus-host interaction networks available at http://pbildb1.univ-lyon1.fr/virhostnet. A total of 16 protein-protein interactions were retrieved and added to our experimental data set.

### Protein-protein interaction Networks

#### Human-human protein-protein interactions network

The 120 human proteins targeted by NS3, NS5 or both flavivirus proteins were linked to form a network of 84 interactions involving 56 proteins by using the reconstructed human-human protein-protein interaction network provided by VirHostNet [19]. All the additional network features presented in the paper were obtained from VirHostNet as well.

#### Visualization

The virus-human and the human-human protein-protein interaction network graphics were performed using the networks GUESS tool http://graphexploration.cond.org.

#### Statistical and topological analysis

All the statistical analyses were performed with the R http://www.r-project.org statistical environment and the igraph R package http://cneurocvs.rmki.kfki.hu/igraph/ was used to compute network metrics.

The degree k of a node v in a graph G is the number of edges that are incident to this node. The betweenness b of a node v in a graph G can be defined by the number of shortest paths going through the node v and is normalized by twice the total number of protein pairs in the graph G (n*(n-1)). The equation used to compute betweenness centrality, b(v), for a node v is:

$$\text{b}\left(\text{v}\right)-\frac{1}{\text{n}\times \left(\text{n}-1\right)}\times \underset{\begin{array}{c} \text{i},\text{j},\text{v}\in \text{V}\\ \text{i}\neq \text{j}\neq \text{v} \end{array}}{\sum }\frac{{\text{g}}_{\text{ij}}\left(\text{v}\right)}{{\text{g}}_{\text{ij}}}$$

where g_ij _is the number of shortest paths going from node i to j, i and j ∈ V and g_ij_(v) the number of shortest paths from i to j that pass through the node v.

### Interconnectivity significance

The overall statistical significance of the interconnectivity (number of protein-protein interactions) between flaviviruses interactors was assessed by a random resampling testing procedure (n = 10, 000 permutations). For each permutation, we randomly extracted as many proteins as the number of flaviviruses interactors from the human interactome, and the value of interconnectivity was assessed. The randomization procedure was weighted and corrected according to the connectivity of proteins in order to prevent inspections bias on highly studied proteins. A theoretical distribution was computed for the 10, 000 resampled values. From this distribution, an empirical p-value for the random resampling test was computed by counting the number of resampled values greater than the observed value.

### Functional analysis using Gene Ontology (GO) annotation

Molecular functions, biological processes and cellular components from Gene Ontology (GO) database [20] were used to annotate the human proteins targeted by the flaviviruses. Briefly, for each GO term, we determine if the set of annotated proteins interacting with the flavivirus proteins is significantly enriched in comparison with the set of proteins annotated with this term within the whole proteome. For each GO term, the enrichment analysis was performed by using an exact Fisher test (p-value < 0.05) followed by the Benjamini and Yekutieli multiple test correction [21]. The analysis was conducted with the web-based software GOEAST [22]

### Sequence identity and similarity between different NS3 helicase proteins

Alignments were performed with the tool « Align » from EMBOSS http://www.ebi.ac.uk/Tools/emboss/align/.

### Cell culture and co-affinity purification

Human HEK-293 null cells were maintained in growth medium consisting of Dulbecco's modified Eagle's medium (DMEM) supplemented with 10% heat-inactivated fetal bovine serum (FBS), 100 U/ml penicillin G, 100 μg/ml streptomycin, at 37°C under 5% CO2.

#### Transient transfection

For all co-affinity purification experiments, HEK-293 cells were transfected with 3 μg of total DNA and 6 μl JetPEI™ transfection reagent according to the manufacturer's instructions (Polyplus Transfection).

#### Co-affinity purification

Two days post transfection, HEK-293 cells were resuspended in lysis buffer (20 mM Tris-HCl at pH 8, 180 mM NaCl, 1% Nonidet P-40, and 2 mM EDTA) supplemented with complete protease inhibitor cocktail (Roche). Cell lysates were incubated on ice for 20 min, and then centrifuged at 14, 000 g for 20 min. 150 μg of protein extracts were incubated for 2 h at 4°C with 50 μl of glutathione-sepharose beads (GE Healthcare) to purify GST-tagged proteins. Beads were then washed 4 times in ice-cold lysis buffer and immuno-precipitated proteins were recovered in loading buffer.

### Western blot

Pull downs and cell lysates (15 μg of protein extracts) were separated by sodium dodecyl sulfate-polyacrylamide gel electrophoresis on 4-12% NuPAGE Bis-Tris gels with MOPS running buffer (SDS-PAGE) (Invitrogen) and transferred to nitrocellulose membrane (I-Blot, Invitrogen). 3XFlag- and GST-tagged proteins were detected with a mouse monoclonal peroxidase-conjugated anti-FLAG M2 antibody (A8592, Sigma) and a rabbit polyclonal anti-peroxidase-conjugated anti-GST antibody (A7340, Sigma) and revealed with ECL detection reagent (pico West, Amersham).

## Results

### Human host proteins targeted by flavivirus replication complex NS3 and NS5 proteins

To unravel new protein-protein interactions between flavivirus and human proteins, we sub-cloned sequences encoding NS3 and NS5 flaviviruses proteins into yeast-two-hybrid (Y2H) vectors. All available viral proteins listed in additional file 1 were expressed in yeast either as full-length proteins or as functional domains, namely NS3 helicase, NS3 protease, NS5 polymerase and NS5 methyltransferase. They were then used as viral baits against human cDNA libraries. Viral ORFs coding for NS3 and NS5 proteins were isolated from distinct human pathogens belonging to major flavivirus evolutionary lineages: *(i) *aedes-borne pathogen: DENV; *(ii) *culex-borne pathogens: WNV (including the Kunjin Australian variant (KUNV)) and JEV; *(iii) *tick-borne pathogens: Tick-borne encephalitis (TBEV) and Alkhurma (ALKV) viruses. Protein sequence comparison study revealed that the functional enzymatic domains of NS3 are highly conserved amongst these viruses (Additional file 2).

At least three independent screenings against human cDNA libraries were performed for each viral bait. Eighty-five percent of the identified cellular targets of each bait were then tested pairwise against all the viral proteins baits including the original bait using an array-based Y2H strategy which confirmed 90% of the interactions identified in the initial screens. Furthermore, the bait panel versus selected targets strategy used in the array cross experiment enabled us to identify 69 additional, novel virus-host interactions not detected in the first screen. Repetition and confirmation of our Y2H experiment by the array strategy allowed us to be very stringent in obtaining a high quality set of 108 human proteins that interacted with one or more of the viral protein baits (Additional file 3). In one of our previously published studies using the same Y2H screening settings, the validation rate obtained by co-affinity purification reached 85% [12]. We conducted GST-pull down assays to further validate our Y2H data (Additional file 4). An extensive literature curation allowed us to finally complete our set of data by 16 previously published interactions, 15 of which not identified by our screen (Additional file 3).

### Analysis of the flavivirus-human protein-protein interaction network

**Figure 1:**
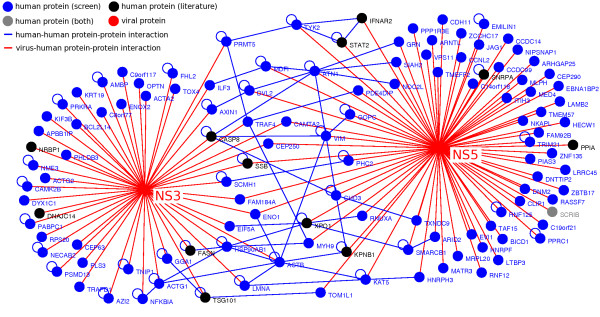
**Human host-flavivirus protein-protein interaction network**. The flavivirus NS3 and NS5 protein interactome, resulting from our Y2H screen and the literature curation, is represented here graphically. Red nodes denote viral proteins; blue nodes denotes human proteins identified by our screen; black nodes are human proteins identified in the literature; gray nodes are human proteins identified both in our screen and in the literature; red edges denote interaction between human and viral proteins; blue edges denote interaction between human proteins. Human proteins interacting with both viral proteins or with other human proteins are positioned centrally.

Based on our high-throughput Y2H screen and literature search, we created the flavivirus NS3 and NS5 proteins interaction network composed of 186 interactions involving 120 distinct human proteins, 108 from our screen and 13 from the literature (Table 1, Figure 1, additional files 3 and 5). We emphasize that among the 186 interactions, 171 were obtained from our Y2H screen and only 16 from previously published work. Despite the conserved amino acid patterns within the different viral ORFs that we used as viral baits, only one third of the cellular targeted proteins identified in our study interacted with two or more flaviviruses (Table 2). Moreover, only five cellular proteins (CAMTA2, CEP250, SSB, ENO1, and FAM184A) were found to interact with both NS3 and NS5 proteins (Figure 1, additional file 5).

**Table 1:** General features of the human host-flavivirus protein-protein interaction network

Origin	Nb of targeted human proteins	Nb of *Flavivirus*-human protein-protein interactions
**Y2H screens**	108	171

**Literature**	13	16

**Y2H screens plus literature**	120	186

General features of the flavivirus network, 1 cellular protein (SCRIB) interacting with TBEV NS5 protein was identified both in the literature and in the Y2H screen.

**Table 2:** Analysis of the human host-flavivirus protein-protein interaction network

Nb of targeting viruses	Nb of targeted human proteins	Targeted human proteins
**4**	2 (1.7%)	APBB1IP, ENO1

**3**	10 (8.3%)	ARID2, AZI2, CAMTA2, CEP63, MLPH, MYH9, NME3, TAF15, TRAF4, VPS11

**2**	26 (21.7%)	ARNTL, BCL2L14, CCDC99, CEP250, DNTTIP2, FAM184A, GGA1, GRN, JAG1, LAMB2, NFKBIA, OPTN, PABPC1, PDE4DIP, PHC2, PHLDB3, PIAS3, RNF125, RNUXA, SCRIB, SNRPA, TOM1L1, TRIM21, TXNDC9, VIM, ZBTB17

**1**	82 (68.3%)	-

We determined the number of flavivirus species that interact with each cellular host protein found to be targeted by NS3 or NS5 (Y2H plus literature).

To further describe the topological properties of the flavivirus interaction network in relation to the whole human interactome, we then took advantage of the VirHostNet knowledgebase which includes an extensive assembly of human-human and viral-human interactions [19]. We thus calculated the local (degree) and global (betweenness) centrality measures of the human proteins targeted by NS3, NS5 or both flavivirus proteins integrated into the human interactome (Table 3). Briefly, the degree of a protein in a network refers to its number of direct partners and is therefore a measure of local centrality. Betweenness is a global measure of centrality, as it measures the number of shortest paths (the minimum distance between two proteins in the network) that cross a given protein. The 120 identified human proteins interacting with NS3 and NS5 were shown to have a higher average degree *i.e*. local connectivity (22, 93 versus 10, 43) and betweenness *i.e*. global centrality (4, 02.10^-4 ^versus 1, 30.10^-4^) in comparison with the human proteins belonging to the human interactome (Table 3). In addition, the degree and the betweenness distributions of human proteins interacting with NS3 and NS5 are significantly distinct from the proteins belonging to the human interactome distributions (U-test, all p-values < 10^-12^, additional file 6). This indicates that NS3 and NS5 have a strong tendency to interact with proteins that are highly connected and central within the human interactome. This latest observation is in accordance with previous virus-host interactome features [11,12,23]. Furthermore, we found that a total of 47 cellular proteins (39%) out of 120 are cellular targets for other viruses as well, including HIV, herpes, hepatitis C and papilloma viruses (Additional file 7, exact Fisher test, p-value = 1, 2.10^-12^). This observation reinforces our findings since different viruses, and possibly other pathogens, are expected to interact with common cellular targets as a consequence of possible common strategies adopted by viruses for infection and replication [23].

**Table 3:** Topological analysis of the human host-flavivirus protein-protein interaction network

Data set	Nb of proteins	Degree	Betweenness (10e-4)
**Human interactome**	10707	10, 43	1.30

**Human proteins targeted** **by NS3 or NS5 of Flavivirus**	108	22.93	4.02

We investigated the topological properties of the 108 connected identified human host proteins in comparison with all the human proteins, which constitute the human interactome. For each dataset, the number of proteins followed by the computed average values of degree and betweenness are given.

### Cellular functions targeted by flavivirus

We then performed an enrichment analysis using Gene Ontology (GO) database on the 120 proteins targeted by the flaviviruses in order to characterize the cellular functions significantly over-represented in the pool of proteins interacting with the flavivirus NS3 and NS5 proteins. Briefly, each cellular protein identified in our analysis and listed in the GO database was ascribed with its GO features. For each annotation term, a statistical analysis evaluated a putative significant over-representation of this term in our list of proteins compared to the complete list of the human annotated proteins. The most significantly over-represented GO annotation terms are listed in Table 4. It is noteworthy that among the enriched functions identified, some are associated with already known function of NS3 and NS5 viral proteins namely RNA binding and viral reproduction (Table 4, molecular function). One may thus put forward the hypothesis that among the cellular proteins listed for these two particular processes some might be key cellular partners for the viral life cycle. We also identified structural components of the cytoskeleton as cellular partners of NS3 and NS5 and we will discuss their putative implication in the viral infectious cycle thereafter in the discussion (Table 4, cellular component). Finally, our analysis revealed that the flaviviruses interact with cellular proteins involved in the Golgi vesicle transport and in the nuclear transport, suggesting that the NS3 and NS5 proteins might be able to interfere with these two cellular functions (Table 4, biological process). In addition, it is important to underline that both regulators of type I interferon-mediated signaling pathway and of innate immune response were found to be significantly enriched in the statistical analysis as well. We will discuss the implication of the functional enrichment profile of the cellular proteins identified in our screen and how these proteins affect the virus replication and assembly.

**Table 4:** Gene Ontology (GO) functional enrichment analysis of the flavivirus-targeted human proteins

Ontology	Description	GO term	p-value	Associated proteins
Molecular function	RNA binding	GO:0003723	****	EIF5A, HNRPF, HNRPH3, ILF3, MATR3, MRPL20, PABPC1, PPRC1, PRKRA, RNUXA, RPS20, SSB, TAF15, TRIM21, SNRPA, XPO1, ZCCHC17
	
	Structural constituent of cytoskeleton	GO:0005200	**	ACTB, ACTG1, BICD1, KRT19, VIM
	
	Nuclear localization sequence binding	GO:0008139	**	KPNB1, NFKBIA
	
	Transcription factor binding	GO:0008134	*	ARNTL, CAMTA2, HNRNPF, KAT5, MDF1, MED4, NFKBIA
	
	Transcription corepressor activity	GO:0003714	*	ATN1, ENO1, RNF12, SIAH2, TSG101

Cellular component	Cytoskeleton	GO:0005856	****	ACTA2, ACTB, ACTG1, ACTG2, APBB1IP, AXIN1, BICD1, CASP8, CCDC99, CEP250, CEP290, CEP63, CHD3, CLIP1, DNM2, FHL2, GOPC, KIF3B, KRT19, LMNA, MLPH, MYH9, PDE4DIP, TRAF4, TYK2, VIM
	
	Ribonucleoprotein complex	GO:0030529	**	ACTB, HNRNPF, HNRNPH3, ILF3, MRPL20, PABPC1, RPS20, SSB, SNRPA, ZCCHC17
	
	H4/H2A histone acetyltransferase complex	GO:0043189	**	ACTB, KAT5

Biological process	Intracellular transport	GO:0046907	***	AXIN1, BICD1, DNM2, EIF5A, GGA1, GOPC, KIF3B, KPNB1, MLPH, NFKBIA, NRBP1, OPTN, RNUXA, TOM1L1, TSG101, XPO1
	
	Regulation of type I interferon-mediated signaling pathway	GO:0060338	***	HSP90AB1, IFNAR2, STAT2, TYK2
	
	Regulation of innate immune response	GO:0045088	**	HSP90AB1, IFNAR2, NFKBIA, TRAFD1, TYK2
	
	Viral reproductive process	GO:0022415	**	KPNB1, PPIA, RPS20, SMARCB1, TSG101, XPO1
	
	Post-Golgi vesicle-mediated transport	GO:0006892	*	DNM2, GOPC, OPTN
	
	Nuclear transport	GO:0051169	*	AXIN1, EIF5A, KPNB1, NFKBIA, RNUXA

We assigned their GO features to the human proteins identified in our screen (literature plus Y2H). We then determined if these features were over-represented in comparison with the complete list of the annotated human proteins. The description of the GO enriched term (column 2), the corresponding GO identifier (column 3), the significativity of the functional enrichment test (**** p-value < = 0.0001, *** p-value < = 0.001, ** p-value < = 0.01, * p-value < 0, 05) and the associated proteins (colum 5) are given in table 4. The three GO subcategories are presented: molecular function, cellular component and biological process.

### Inter-connection of the cellular proteins targeted by flaviviruses

Only 1/3 of the cellular proteins are represented in the human-human protein-protein interactome, suggesting that most of the cellular proteins are not connected [19]. We observed that the human proteins targeted by the flavivirus NS3 and NS5 were highly overrepresented in the human interactome (108/120, exact Fisher test, p-value < 2, 2.10^-16^). This implies that most of the cellular proteins targeted by the flaviviruses are connected with other human proteins. An analysis of the level of interconnectivity of the 108 proteins revealed that they are indeed highly connected to each other (84 protein-protein interactions), and that this interconnectivity is highly significant compared to the theoretical interconnectivity computed from resampled networks (resampling test, n = 10, 000, p-value < 10^-4^, additional file 8). All together these results, in accordance with our functional enrichment analysis, emphasized the fact that the flaviviruses are targeting closely related cellular proteins, which are likely to share common functional features.

**Figure 2:**
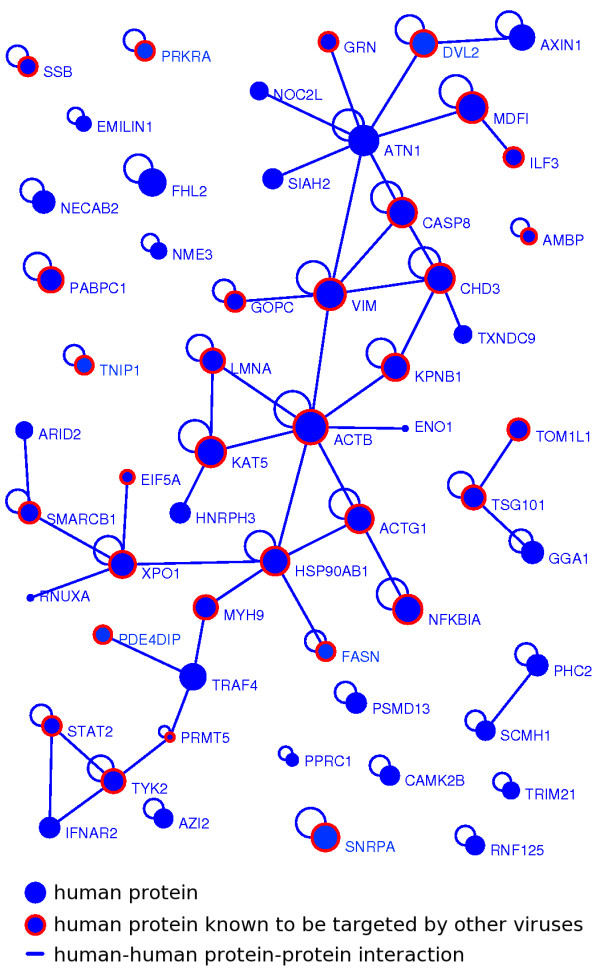
**Flavivirus targeted human protein-protein interaction sub-network**. The human host proteins interacting with the NS3 or the NS5 viral proteins form a connected sub-network represented here graphically. Blue nodes denote human proteins; blue edges interaction between human proteins; red strokes denote human proteins targeted by at least one protein from another virus than Flavivirus. The width of the nodes is roughly proportional to the cellular degree, *i.e*. the number of cellular partners in the whole human network. The largest component containing 35 proteins is represented in the middle of the network.

Figure 2 represents the sub-network of all the cellular proteins connected into the human protein-protein network and targeted by the flavivirus replication complex NS3 or NS5 proteins. These interacting proteins form a relatively compact connection web with a central core of 35 proteins, the majority of which has been shown to interact with other viruses (Figure 2 and additional file 7). Interestingly, among these central proteins, several are important components of the cytoskeleton. These include in particular VIM, MYH9, ACTB, ACTG1, LMNA and GOPC (Table 2). NS3 and NS5 are interacting with two smaller functional units: one is composed by 4 proteins belonging to the interferon signalling cascade (PRMT5, TYK2, STAT2 and IFNAR2) and the second one is made up by 3 molecules involved in vesicular transport (TSG101, GGA1 and TOM1L1).

## Discussion

Among the 53 species of flavivirus, 40 are associated with potentially life-threatening human infections. Due to the rapid expansion of arthropod vectors and the limited number of existing vaccines (*i.e*. against YFV, JEV and TBEV), the understanding of flavivirus pathogenesis represents a major challenge in public health research. In particular, deciphering the interactions between flavivirus proteins and human host proteins may prove to be of great value for designing new vaccines or curative treatments targeting human cellular factors rather or in complement to viral targets. To achieve this goal, different innovative experimental approaches that rely on systemic biology were recently developed [14]. Using a high-throughput yeast two-hybrid screening strategy, we report here the identification of more than 100 novel human proteins directly interacting with the flavivirus replication complex proteins, NS3 or NS5. These newly identified cellular partners considerably expand the number of host proteins being potentially involved at some point in the flavivirus life cycle. It is worth noting that most of the cellular proteins identified here have not been previously reported in the literature as flavivirus host factors, including in the two recent genome-wide RNA interferences studies [15,16] and a DENV2 bacterial two-hybrid screen [24]. This lack of redundancy, which is commonly reported for such large-scale studies, implicates that both RNAi and two-hybrid approaches are not exhaustive and that complementary experimental approaches are needed to construct a comprehensive scheme of virus-host interactions eventually [25]. Interestingly, the topological analysis of our flavivirus-human protein-protein interaction network reveals that flaviviruses interact with highly connected and central cellular proteins of the human interactome, as previously reported for the hepatitis C Virus (HCV) and the Epstein Barr Virus (EBV) [11,12]. Our study also unravels numerous shared cellular targets between flaviviruses and the Human Immunodeficiency Virus (HIV), the Papilloma viruses and the Herpes viruses. This finding supports the idea that a large variety of viruses use common mechanisms to interfere with cell organisation.

Besides providing a synthetic view of flavivirus-host interactions, our interactome study sheds new light on the pathogenesis of flavivirus infections. In particular, the NS3 and NS5 viral proteins were found to interact with several cellular proteins involved in histone complexe formation and/or in the chromatin remodelling process namely CHD3, EVI1, SMARCB1, HTATIP, and KAT5. Similarly in a recent system biology study aimed at describing the mammalian transcriptional network in dendritic cells, Amit *et al*. proposed that the chromatin modification may be a key event during dendritic cells immune response against pathogens [26]. Interestingly, dengue virus presents a high primary tropism toward cells of the phagocyte mononuclear system, namely dendritic cells of the skin (Langerhans cells), monocytes and macrophages. Thus, the fact that proteins belonging to the flavivirus replication complex directly target central components of histone complex might suggest that flaviviruses escape host defense by disrupting and/or subverting the control of chromatin organization within infected immune cells. Moreover, by interacting with the chromatin remodelling machinery, some flaviruses may take advantage of host cells' replicative machinery to interfere with the host cellular homeostasis and/or to replicate their own genome as previously shown for SMARCB1 and retroviral genome replication [27]. However, knowing that most flaviviruses replicate their genome in association with host cell membranes in the perinuclear region of the cytoplasm, the hypothesis of a chromatin-dependent replication is unlikely. Indeed, even though DENV-2 NS5 contains two functional NLS which were shown to interact with the importin and the exportin proteins, KPNB1 and XPO1 [28,29], the role of NS5 in the nucleus has not yet been elucidated [6]. The NS3 and NS5 proteins were also found to interact with several proteins belonging to the cell RNA processing machinery such as HNRPF, PABPC1 or HNRPH3. These results are in accordance with the recent identification of non-polyadenylated 3' end of dengue virus RNA as a viral partner for PABPC1 [30] and emphasize the possible cooperation between viral and human proteins during viral genome replication.

A common feature observed in a large number of viruses is their ability to disorganize the cytoskeleton by targeting central component of the microtubule, intermediate or micro-filament system networks. In this regard, our data are in accordance with a genome-scale RNAi screen which revealed that silencing genes involved in intracellular trafficking affects the outcome of a WNV infection [16]. However, our work not only demonstrates that flavivirus proteins interact with cytoskeleton components known to be targeted by other viruses but also identifies new host protein targets involved in intracellular trafficking. These include in particular the kinesin family member KIF3B and the centrosomal components CEP63, CEP250 and CEP290. ACTB and VIM appear as central "hubs" in the highly connected flavivirus-human protein network suggesting they may be key components of viral particle production. Supporting this view, dengue virus production has already been associated with vimentin filament perturbation [31]. Besides proteins involved in cytoskeleton network, we also identified a smaller sub-network composed of three proteins belonging to the post-Golgi vesicular transport (TOM1L1, TSG101 and GGA1) and four proteins associated with the Golgi vesicle transport (DNM2, GOPC, NRBP1, OPTN). These proteins are most likely involved in the virus-induced membrane rearrangements associated to DENV replication and assembly in the so-called replication factories [7,32].

## Conclusion

In conclusion, we report here the results of a proteome mapping screen to identify the interactions between human host proteins and the flavivirus NS3 and NS5 proteins. Our high-throughput yeast two-hybrid screen identified 108 human proteins interacting with NS3 or NS5 proteins or both. And our virus-host interaction map provides a foundation to unravel fundamental processes about flavivirus subversion of the host replication machinery and/or the immune defence strategy of the host.

## Competing interests

The authors declare that they have no competing interests.

## Authors' contributions

MLB carried out the Y2H screen and the molecular cloning of the viral ORFs. LMS performed all the statistical and bio-informatic analyses; she also helped to draft the manuscript. AD participated in the Y2H screen and the molecular cloning of the viral ORFs. BCo participated in the molecular cloning of the viral ORFs and helped to draft the manuscript. BCa, XdeL participated in the design and the coordination and helped to draft the manuscript. PA, CRC and VL conceived the original mapping project. ND coordinated the project and drafted the manuscript. All authors read and approved the final manuscript.

## Supplementary Material

Additional file 1 **Description of all the viral baits used in the Y2H screen**. The viral baits are identified by their ViralORFeome identifier (column 2) and their associated GenBank protein identifier (column 3). Length, coordinates in the coding sequence and mutations are listed in ViralORFeome database http://www.viralorfeome.com. Click here for file

Additional file 2 **The NS3 helicases sequences identity and similarity**. For each protein pair, an alignment was performed and the protein sequence identity (blue) and similarity (black) percentage were given. Bold values represent high values of identities or similarities. Click here for file

Additional file 3 **List of the human proteins identified as flavivirus NS3 or NS5 targets**. Flavivirus NS3- or NS5-targeted human proteins are referenced by their HGNC symbol (column 1) and their Ensembl Gene ID (column 2), their Ensembl description (column 3) and their source: Y2H screen (column 4) and/or literature (column 5). Click here for file

Additional file 4 **Validation of three Y2H interactions showing that DENV 2 NS3 interacts with some proteins involved in the innate immune response**. HEK-293T cells were co-transfected with expression vectors encoding the GST alone or the GST fused to DENV2 NS3 helicase, and 3xFlag tagged TRAF4, NFKBIA or AZI2. Co-purifications were obtained by pull-down on total cell lysates. GST-tagged viral NS3 proteins were detected by immuno-blotting using anti-GST antibody, while TRAF4, NFKNIA or AZI2 were detected with anti-Flag antibodies before (lower panel, cell lysate) and after pull-down (upper panel, pull down). Click here for file

Additional file 5 **Human host-flavivirus NS3 and NS5 protein-protein interactions, functional domains specification**. Human proteins are referenced by their HGNC symbol (column 1) and their Ensembl Gene ID (column 2), and the characteristics of the viral proteins are reported in column 3. The origin of the interaction is indicated in column 4 (Y2H screens) and/or 5 (literature). Click here for file

Additional file 6 **Degree and betweenness distributions**. Degree (left) and betweenness [29] distributions of human proteins (black) and human proteins targeted by flavivirus proteins (red) in the human interactome. P(k) is the probability of a node to connect k other nodes in the network. P(b) is the probability of a node to have a betweeness equal to b in the network. Solid lines represent the linear regressions. Vertical dashed lines give mean degree and betweenness values. Click here for file

Additional file 7 **Flavivirus-targeted human proteins interactions with other viral proteins**. Human proteins are referenced with their Ensembl Gene ID (column 1) and their HGNC symbol (column 2), viral proteins with their virus name (column 3), their NCBI id (column 4) and their NCBI name (column 5). These data were collected from the VirHostNet knowledge base. Click here for file

Additional file 8 **Statistical analysis of the interconnectivity of the human interactors of NS3 and NS5**. Numbers distribution of protein-protein interactions was obtained by random simulation. 108 genes were randomly drawn from the genome 10, 000 times, and the 10, 000 numbers of protein-protein interactions in the subgraph existing between theses genes were plotted. A vertical arrow indicates the observed value of 84 interactions with its significance. Click here for file
