# Supplementary material for: Flavivirus NS3 and NS5 proteins interaction network: a high-throughput yeast two-hybrid screen
Source: BMC Microbiol. 2011 Oct 20;11:234. doi: 10.1186/1471-2180-11-234 (PMC3215679; doi:10.1186/1471-2180-11-234)
Supplement: Additional file 4 — Validation of three Y2H interactions showing that DENV 2 NS3 interacts with some proteins involved in the innate immune response. HEK-293T cells were co-transfected with expression vectors encoding the GST alone or the GST fused to DENV2 NS3 helicase, and 3xFlag tagged TRAF4, NFKBIA or AZI2. Co-purifications were obtained by pull-down on total cell lysates. GST-tagged viral NS3 proteins were detected by immuno-blotting using anti-GST antibody, while TRAF4, NFKNIA or AZI2 were detected with anti-Flag antibodies before (lower panel, cell lysate) and after pull-down (upper panel, pull down). [file 1471-2180-11-234-S4.PPT]

## Slide 1
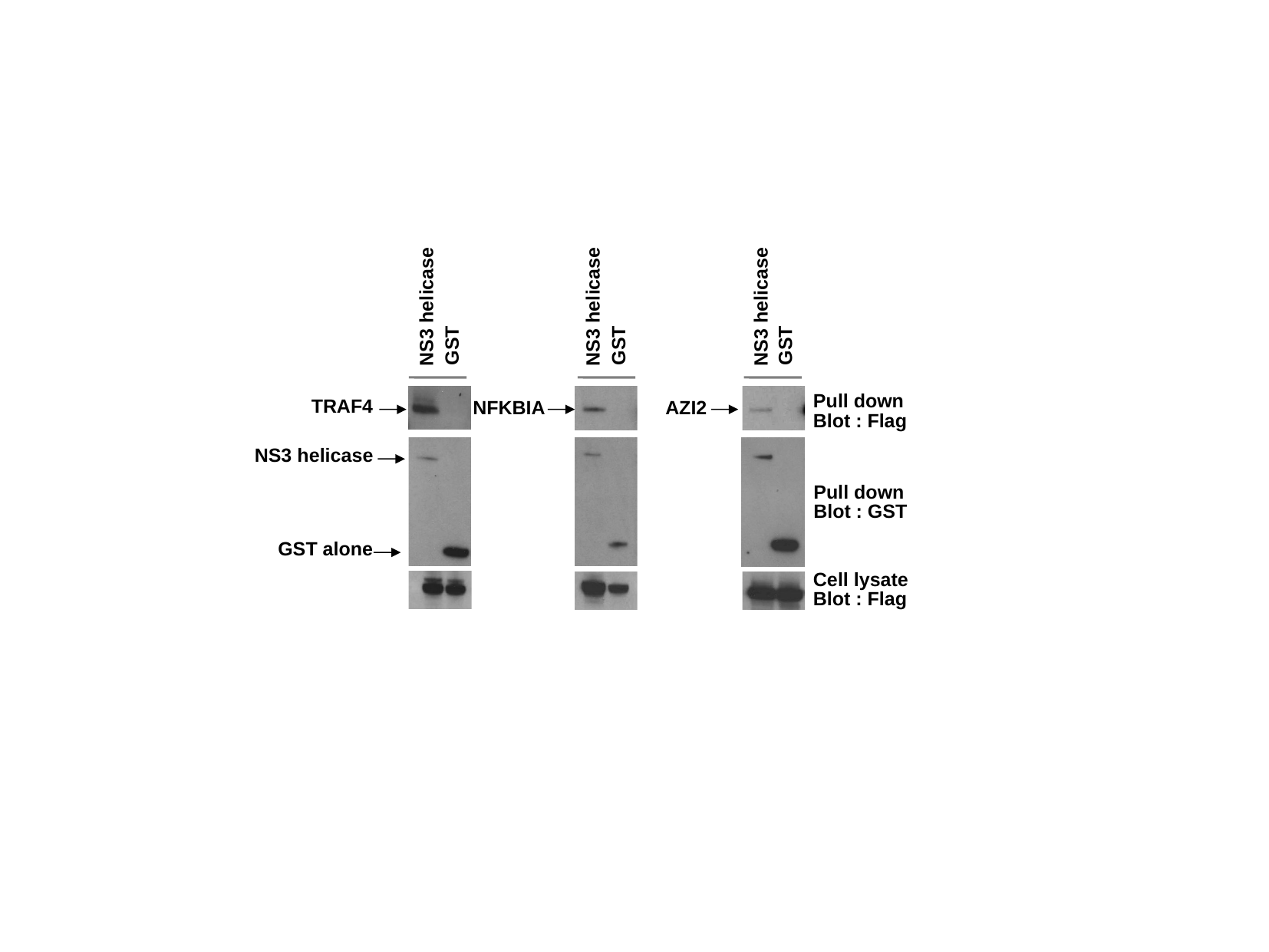

NS3 helicase
NS3 helicase
NS3 helicase
GST
GST
GST
Pull down
Blot : Flag
TRAF4
 NFKBIA
AZI2
NS3 helicase
Pull down
Blot : GST
GST alone
Cell lysate
Blot : Flag
