# Supplementary figures and images for: Flavivirus NS3 and NS5 proteins interaction network: a high-throughput yeast two-hybrid screen
Source: BMC Microbiol. 2011 Oct 20;11:234. doi: 10.1186/1471-2180-11-234 (PMC3215679; doi:10.1186/1471-2180-11-234)

## Slide 1
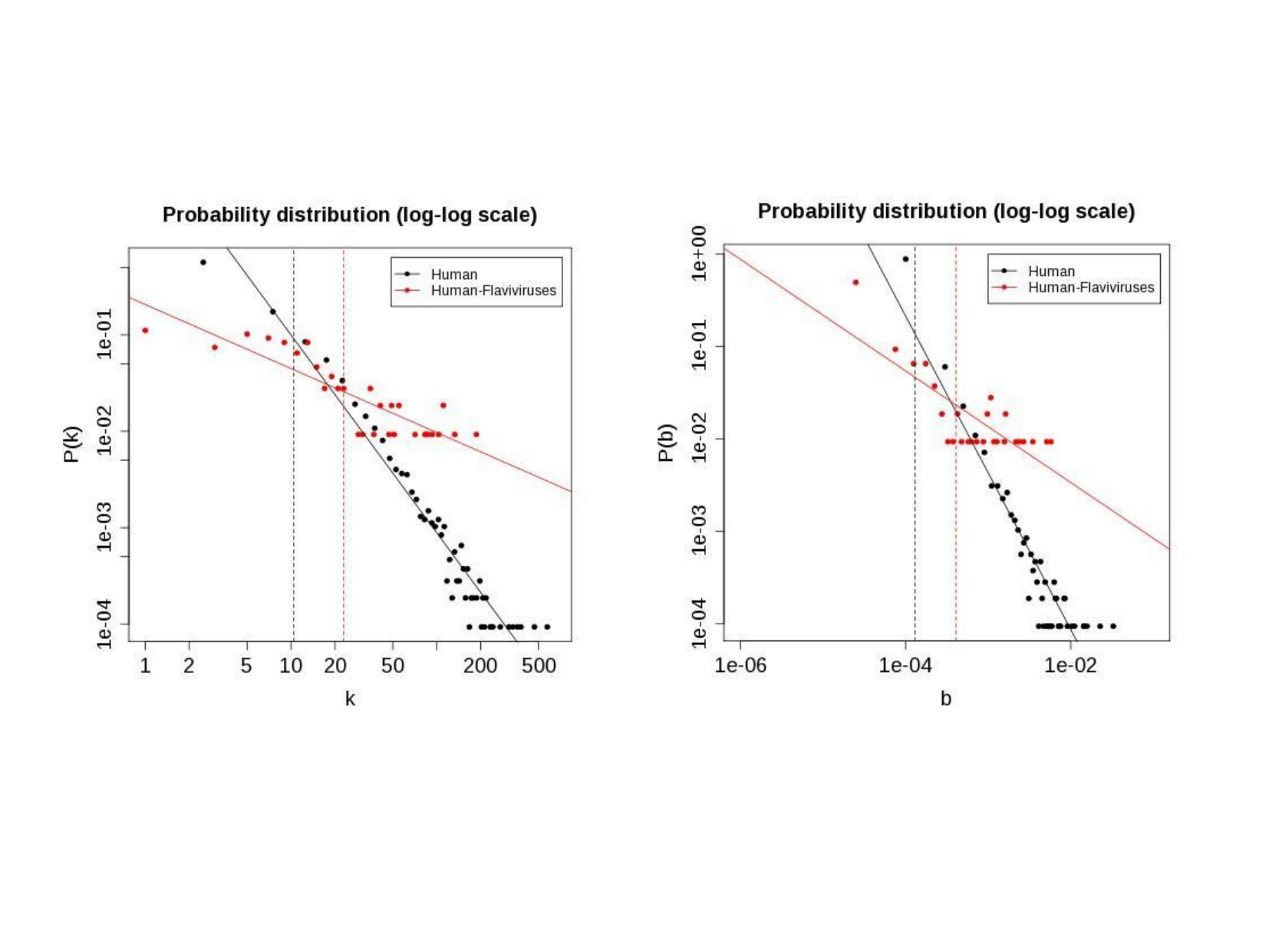

Supplement: Additional file 6 — Degree and betweenness distributions. Degree (left) and betweenness [29] distributions of human proteins (black) and human proteins targeted by flavivirus proteins (red) in the human interactome. P(k) is the probability of a node to connect k other nodes in the network. P(b) is the probability of a node to have a betweeness equal to b in the network. Solid lines represent the linear regressions. Vertical dashed lines give mean degree and betweenness values. [file 1471-2180-11-234-S6.PPT]

## Slide 1
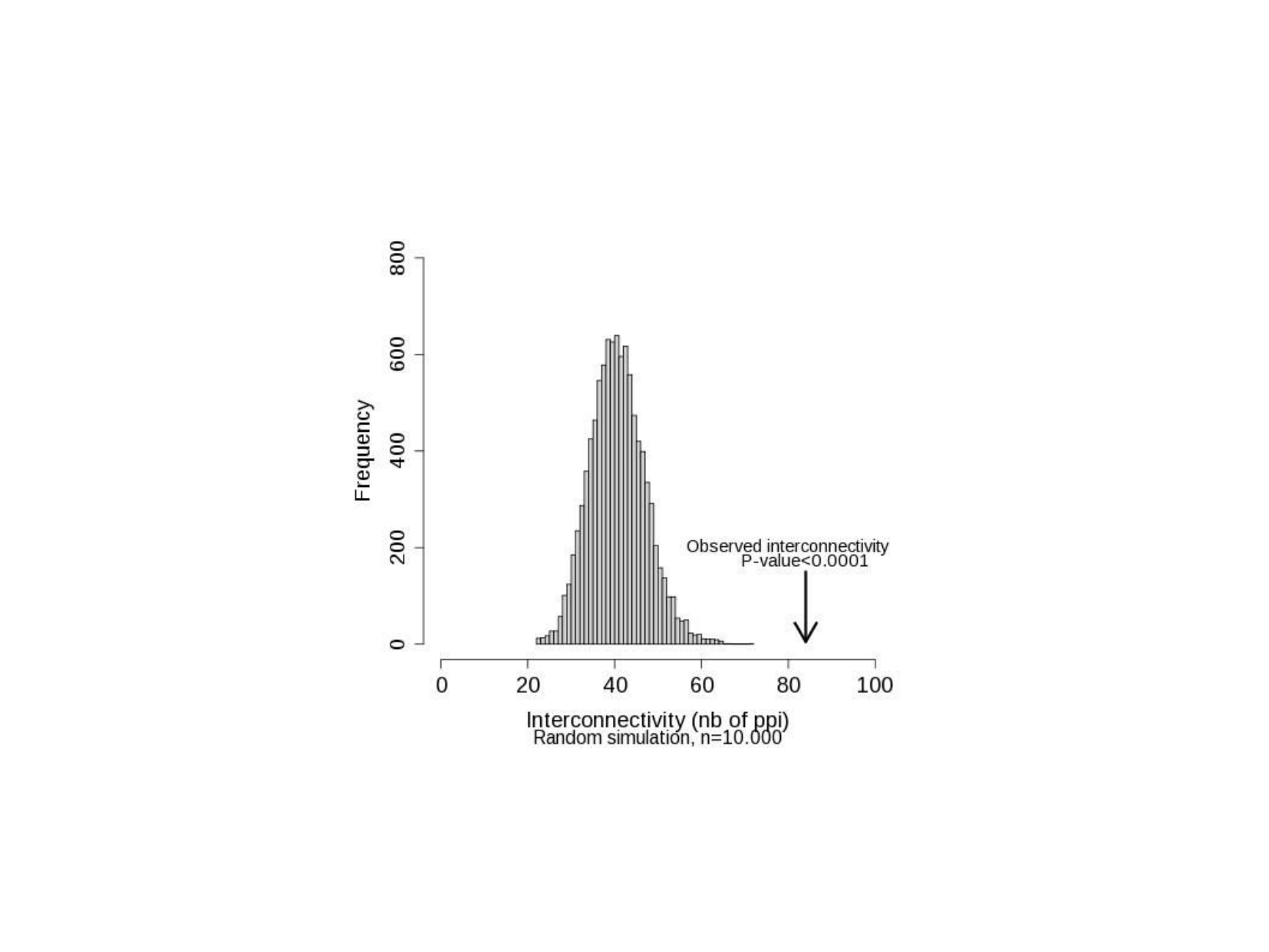

Supplement: Additional file 8 — Statistical analysis of the interconnectivity of the human interactors of NS3 and NS5. Numbers distribution of protein-protein interactions was obtained by random simulation. 108 genes were randomly drawn from the genome 10, 000 times, and the 10, 000 numbers of protein-protein interactions in the subgraph existing between theses genes were plotted. A vertical arrow indicates the observed value of 84 interactions with its significance. [file 1471-2180-11-234-S8.PPT]
